# Rutin Nanocrystals with Enhanced Anti-Inflammatory Activity: Preparation and Ex Vivo/In Vivo Evaluation in an Inflammatory Rat Model

**DOI:** 10.3390/pharmaceutics14122727

**Published:** 2022-12-06

**Authors:** Abeer S. Hassan, Ghareb M. Soliman

**Affiliations:** 1 Department of Pharmaceutics, Faculty of Pharmacy, South Valley University, Qena 83523, Egypt; 2 Department of Pharmaceutics, Faculty of Pharmacy, University of Tabuk, Tabuk 71491, Saudi Arabia; 3 Department of Pharmaceutics, Faculty of Pharmacy, Assiut University, Assiut 71526, Egypt

**Keywords:** rutin, nanocrystals, anti-inflammatory, hydroxypropyl beta-cyclodextrin, nanoparticles

## Abstract

Rutin is a polyphenolic flavonoid with an interestingly wide therapeutic spectrum. However, its clinical benefits are limited by its poor aqueous solubility and low bioavailability. In an attempt to overcome these limitations, rutin nanocrystals were prepared using various stabilizers including nonionic surfactants and nonionic polymers. The nanocrystals were evaluated for particle size, zeta potential, drug entrapment efficiency, morphology, colloidal stability, rutin photostability, dissolution rate, and saturation solubility. The selected nanocrystal formulation was dispersed in a hydrogel base and the drug release kinetics and permeability through mouse skin were characterized. Rutin’s anti-inflammatory efficacy was studied in a carrageenan-induced rat paw edema model. The nanocrystals had a size in the range of around 270–500 nm and a polydispersity index of around 0.3–0.5. Nanocrystals stabilized by hydroxypropyl beta-cyclodextrin (HP-β-CD) had the smallest particle size, highest drug entrapment efficiency, best colloidal stability, and highest drug photostability. Nanocrystals had around a 102- to 202-fold and 2.3- to 6.7-fold increase in the drug aqueous solubility and dissolution rate, respectively, depending on the type of stabilizer. HP-β-CD nanocrystals hydrogel had a significantly higher percent of drug released and permeated through the mouse skin compared with the free drug hydrogel. The cumulative drug amount permeated through the skin was 2.5-fold higher than that of the free drug hydrogel. In vivo studies showed that HP-β-CD-stabilized rutin nanocrystals hydrogel had significantly higher edema inhibition compared with the free drug hydrogel and commercial diclofenac sodium gel. These results highlight the potential of HP-β-CD-stabilized nanocrystals as a promising approach to enhance drug solubility, dissolution rate, and anti-inflammatory properties.

## 1. Introduction

Rutin, also known as vitamin P, is a polyphenolic flavonoid found in plants such as buckwheat, green tea, citrus fruits (e.g., orange, grapefruit, lemon, lime), and apples [1,2]. Chemically, rutin is known as (2-(3,4-dihydroxyphenyl)-4,5-dihydroxy-3-[3,4,5-trihydroxy-6-[(3,4,5-trihydroxy-6-methyl-oxan-2-yl)oxymethyl]oxan-2-yl]oxy-chromen-7-one) or quercetin-3-rutinoside. Rutin has several attractive features as a drug such as its natural source, safety, cost-effectiveness, and wide spectrum of pharmacological actions [3,4,5]. Moreover, several reports have shown rutin to have antioxidant, antidiabetic, anticancer, anti-inflammatory, antibacterial, anti-arthritic, and neuroprotection activities [5,6,7,8]. In addition, rutin was also shown to have antihypertensive, cardioprotective, antispasmodic, anti-thrombotic, and anti-hyperlipidemia actions [9,10,11,12]. Despite these interesting pharmacological properties, rutin suffers from some drawbacks such as poor aqueous solubility and stability which result in poor oral bioavailability [13,14]. This poses some challenges for the formulation of oral dosage forms of rutin whether for its application as a nutritional supplement or a therapeutic agent [2]. To overcome these shortcomings and improve rutin’s therapeutic efficacy, several formulation strategies and drug delivery systems have been exploited. For instance, dried rutin nanocrystals have been prepared and incorporated into tablets [2]. The nanocrystal tablets achieved complete rutin dissolution in 30 min compared to only 71% and 55% dissolution from the microcrystal tablets and the commercialized tablets, respectively. Rutin-loaded silver nanoparticles were fabricated and their anti-thrombotic activity was evaluated [12]. Rutin-silver nanoparticles had prolonged activated partial thromboplastin time and prothrombin time. Quinoa and maize starch nanoparticles were also used to encapsulate rutin and improve its bioavailability [15]. In a simulated in vitro digestion test, the nanoparticles were able to increase rutin’s bioavailability and improve its antioxidant activity. Rutin was also loaded into liquid crystalline nanoparticles (LCNs) and its anticancer activity against non-small cell lung cancer was tested in an A549 human lung epithelial carcinoma cell line [16]. Rutin-LCNs showed promising anti-proliferative and anti-migratory activities. In addition, they induced apoptosis in the A549 cells and inhibited colony formation.

Nanocrystals (NCs) are sub-micron colloidal dispersions composed of 100% drug material. They were first introduced in the 1990s as a means of improving dissolution rate, aqueous solubility, and thus the bioavailability of poorly water-soluble drugs [17]. NCs have a particle size in the nanometer range and are typically produced by the milling of bulk drug material [18]. They are stabilized by surfactants, polymers, or both [19]. The enhanced dissolution rate and solubility are believed to be due to the decrease in particle size, which in turn increases the surface area available for dissolution according to the Noyes–Whitney equation [20]. The enormous increase in surface area and saturation solubility result in improved drug oral bioavailability and permeability through biological membranes making them an attractive drug delivery approach for poorly water soluble drugs [18,19,21]. These interesting features culminated in the approval of Rapamune^®^ (sirolimus, Pfizer), Megace ES^®^ (megestrol acetate, Elan/Par Pharm), Emend^®^ (aprepitant, MSD), Tricor^®^ (fenofibrate, AbbVie), and Invega Sustenna^®^ (paliperidone palmitate, Elan/Johnson and Johnson) for oral administration [18,22]. Nanocrystals have also found interesting applications for dermal and transdermal drug delivery since they were reported to increase drug penetration through the skin [18,23]. This is usually achieved by virtue of nanocrystals’ ability to create a higher concentration gradient across the skin due to increased drug saturation solubility leading to increased passive diffusion [24]. They also increase drug delivery through the hair follicles due to particle size reduction, as well as adhesion to the skin [23]. However, no nanocrystal-based topical products have been approved for clinical use so far thus showing that further research is still needed in this area.

The literature shows a very limited number of reports on using nanocrystals to improve the dermal and transdermal delivery of rutin. Thus, Pelikh et al. prepared rutin and hesperetin nanocrystals with a size in the range of 160–700 nm and studied the effect of particle size and vehicle type on the skin penetration of the drugs [18]. The results showed that oleogels and creams were better than hydrogels in improving rutin nanocrystals skin penetration. In addition, smaller particles achieved better skin penetration. Furthermore, Pyo et al. showed that particle size also influenced the antioxidant efficacy of rutin nanocrystals where the nanocrystals with a mean dimeter of 300 nm had the highest antioxidant capacity compared to the drug microparticles (33 µm) and commercial cosmetic drug products [25]. In another study, rutin nanocrystals were suspended in Carbopol gel and tested as an anti-photoaging agent [26]. The nanocrystal gel achieved 3-fold higher drug permeability through mice skin compared with the coarse drug gel and prevented UV irradiation-induced photoaging and tissue damage. For cosmetic applications, rutin nanocrystal formulation is commercially available under the tradename Juvedical^®^ (Juvena) [27].

To date no reports, however, have studied the effects of nanocrystal formulation on rutin’s anti-inflammatory properties following topical application. Therefore, the aim of this study was to prepare rutin nanocrystals using different stabilizers such as Pluronic F-17, Tween 80, hydroxypropyl beta-cyclodextrin, and PEG 6000 and test their ability to enhance rutin’s permeability through mice skin. In addition, the in vivo anti-inflammatory properties of the selected rutin nanocrystal formulations were examined using a carrageenan-induced rat paw edema model.

## 2. Materials and Methods

Rutin (RT) (purity > 95%) was purchased from Oxford Lab Fine Chem LLP, Vasai, India. Hydroxypropyl beta-cyclodextrin (HP-β-CD), and Pluronic F-127 was obtained from Sigma-Aldrich (St. Louis, MO, USA). Potassium dihydrogen phosphate, PEG 200, PEG 6000, Tween 80, hydroxypropyl methyl cellulose (HPMC), disodium hydrogen phosphate, and sodium hydroxide were obtained from United Company for Chem. and Med. Prep., Cairo, Egypt.

### 2.1. Preparation of Rutin Nanocrystals (RT-NCs)

RT-NCs were prepared by modifying the anti-solvent nanoprecipitation–ultrasonication method reported in the literature [28]. ln brief, RT was dissolved in ethanol to prepare the organic phase. The anti-solvent phase was prepared by dissolving the stabilizers (Pluronic F-17, Tween 80, HP-β-CD, or PEG 6000) at a concentration of 0.2%, *w/v* in distilled water with 1 mL of PEG 200 as a co-stabilizer (Table 1). The organic phase was added drop-wise by a syringe into the specified volume of anti-solvent phase and the dispersion was stirred on a magnetic stirrer at a speed of 3000 RPM for 2 h at room temperature to remove the organic solvent. The obtained suspension was subjected to ultrasonication using a probe sonicator (Cole-Parmer, Vernon Hills, IL, USA) at 5 s pauses, 5 s ON at an amplitude of 45% to form nanosized particles [29]. A drug suspension without a stabilizer was prepared for comparison studies.

### 2.2. Particle Size, Polydispersity Index, and Zeta Potential Measurements

The mean particle size, polydispersity index (PDI), and zeta potential were determined by dynamic light scattering using a Malvern Zetasizer Nanoseries ZS^®^ instrument (Malvern Instruments, Malvern, UK) equipped with a backscattered light detector operating at 173°. The measurements were performed in triplicate at room temperature.

### 2.3. Determination of Percent Drug Entrapment Efficiency (%EE)

The entrapment efficiency of RT nanocrystals was evaluated indirectly by estimating the unentrapped RT. Briefly, the unentrapped drug was separated from nanocrystals by centrifugation at 15,000 RPM for 30 min at 4 ± 0.5 °C using cooling ultracentrifuge. The concentration of the drug in the supernatant was measured spectrophotometrically at λ_max_ of 359 nm (LISCO GmbH, Bargteheide, Germany) [30] and using a calibration curve (y = 0.0151x + 0.1129, where y is the absorbance and x is rutin concentration, *R*^2^ = 0.9992). The drug entrapment efficiency (%EE) was determined by applying the following equation:

(1) $$\mathrm{EE}\left(\%\right)=\frac{\left(\mathrm{Total}\;\mathrm{drug}\;-\mathrm{Drug}\;\mathrm{in}\;\mathrm{supernatant}\;\right)}{\mathrm{Total}\;\mathrm{drug}\;}\times 100\;\;$$

### 2.4. Stability Studies for RT-NCs

#### 2.4.1. Assessment of RT-NCs Physical Stability

RT-loaded NC dispersions prepared with different stabilizers were stored in a dark place at an ambient temperature for up to three weeks. The physical stability of RT-NC formulations was evaluated by visual appearance and settlement volume ratio (*F*). The settlement volume ratio is the ratio of volume or height before and after sedimentation for a given period [31,32]. It was calculated using the following equation:

(2) $$F=\;\frac{V}{V_{0}}=\frac{H}{H_{0}\;}$$

where *H*_0_ is the height of suspension before sedimentation and *H* is the height of sediment surface after sedimentation. *V* and *V*_0_ are the suspension volumes after and before sedimentation, respectively.

#### 2.4.2. Storage Stability

An aqueous dispersion of the selected formulation (RT-NC2) was transferred into sealed brown glass bottles and stored at two different storage conditions: the room temperature and refrigerated conditions of (4 °C) for 60 days. At various time intervals, aliquots were withdrawn and analyzed for their particle size, polydispersity index, and percent drug entrapment efficiency.

#### 2.4.3. Storage Chemical Photostability

Free RT and RT-NC2 aqueous dispersions were transferred into transparent glass vials and sealed by rubber stoppers. The vials were exposed to sunlight for 1 month at room temperature. At various time intervals, aliquots were withdrawn and methanol was added to dissolve RT followed by filtration to remove undissolved materials [33]. The RT concentration in the filtrate was measured spectrophotometrically at 359 nm.

The percent of RT remaining after different light exposure times was calculated using the following equation:

(3) $$\mathrm{RT}\;\mathrm{remaining}\;\left(\%\right)=\frac{C_{t}}{C_{0}}\times 100\;\;\;\;\;\;$$

where *C*_0_ and *C_t_* are the concentrations of RT at zero time and various time intervals, respectively.

### 2.5. Lyophilization of RT-NCs

RT-NCs were lyophilized to convert them to a dry form. The formulations were transferred to glass flasks then frozen over night at −80 ± 1 °C and lyophilized over a period of 48 h using a FreeZone freeze drier (Labconco Inc., Kansas City, MO, USA). The dried nanocrystals were stored in a desiccator until further investigations.

### 2.6. Characterization of Freeze-Dried Powder of RT-NCs

#### 2.6.1. Fourier Transform Infrared Spectroscopy (FT-IR) Studies

The FT-IR spectra of RT alone, HP-β-CD alone, their physical mixture (1:1, *w*/*w*), and selected RT–nanocrystal formulation (RT-NC2) were recorded using a Shimadzu IR-470 spectrophotometer (Shimadzu, Seisakusho Ltd., Kyoto, Japan) at a wavenumber range of 4000–400 cm^−1^. The potassium bromide (KBr) disc method was used. The samples were ground, mixed thoroughly with KBr, and compressed into discs using an IR compression machine.

#### 2.6.2. Saturation Solubility

An excess amount of sample (equivalent to 5 mg) was placed in a screw-capped glass vial containing 500 mL of phosphate buffer pH 6.5 and shaken in a thermostatically controlled shaking water bath (DAIHAN Scientific Co., Seoul, Republic of Korea) at 50 RPM and 37 °C for 48 h until equilibrium was attained [34]. The suspensions were filtered using a membrane disc filter (0.45 µm) and the drug concentration in the filtrate was determined spectrophotometrically at λ_max_ of 359 nm.

#### 2.6.3. In Vitro Drug Dissolution Studies

Dissolution studies were performed in pH 6.5 phosphate buffer containing 0.25% *v*/*v* ethanol using an USP XXIV type II dissolution apparatus. Free RT and freeze-dried RT-NCs were dispersed into 500 mL of the dissolution medium and stirred at 50 RPM at 37 ± 0.5 °C. At various time intervals (0, 5, 15, 30, 60, 90, and 120 min), an aliquot (5 mL) was withdrawn and replaced immediately with the same volume of fresh dissolution medium. The drug concentration in the withdrawn samples was measured spectrophotometrically at 359 nm. The dissolution experiments were conducted in triplicate.

#### 2.6.4. Scanning Electron Microscopy (SEM) Studies

The surface morphology of RT-NCs was examined using scanning electron microscopy (SEM) (Jeol, JSM-5200, Tokyo, Japan). A sample of selected formulation (RT-NC2) was prepared by applying a droplet of RT-NCs onto an aluminum specimen stub, dried overnight, and sputter-coated with gold prior to imaging. An acceleration voltage of 15 kV was utilized.

### 2.7. Preparation and Characterization of Free RT and RT-NCs Hydrogels

An aqueous warm dispersion of a weighed amount of hydroxypropyl methyl cellulose (HPMC 15000) (5%, *w*/*w*) was developed with continuous stirring until plain gel was formed. This concentration of HPMC was selected based on previous work to produce hydrogels with desirable viscosity and homogeneity. The dispersion was sonicated for 15 min to remove air bubbles. RT dispersion in distilled water was added slowly to 10 mL of aqueous HPMC dispersion while stirring took place until a homogenous RT hydrogel was formed. The calculated amount of freeze-dried selected RT-NC2 formulation was incorporated into HPMC plain gel 5%, *w*/*v* by magnetic stirring and the final weight of the gel was adjusted to 10 g with distilled water. The RT concentration in the free RT and RT-NC2 hydrogels was 0.5%, *w*/*w*. The prepared free RT and RT-NC2 hydrogels were left in the fridge for further studies. The viscosity of the hydrogel was measured by a Brookfield Digital Viscometer (Model DV-II Brookfield Engineering Laboratories, Inc., Stoughton, MA, USA). The pH of the free RT and RT-NC2 hydrogels was measured using a pH meter (3500 pH meter, Jenway, UK). The RT content of the hydrogels was measured by dissolving 0.5 g in methanol and the drug concentration was measured spectrophotometrically at λ_max_ of 359 nm.

### 2.8. In Vitro Drug Release Studies from Hydrogels

The release of RT from free RT and RT-NC2 hydrogels was characterized using the dialysis method through a semi-permeable cellophane membrane (molecular weight cutoff 12,000–14,000, Sigma Aldrich, St. Louis, MO, USA) as mentioned previously with slight modification [35]. Briefly, the tested formulation (1 g of hydrogel equivalent to 5 mg RT) was placed over a previously soaked cellophane membrane fitted at the bottom of a glass tube open at both sides. The glass tube was immersed in a beaker containing 100 mL of phosphate buffer pH 6.5 with 0.25%, *v*/*v* ethanol. The beakers were placed in a thermostatically controlled shaking water bath, (DAIHAN Scientific Co., Seoul, South Korea) operating at 50 RPM and 37 ± 0.5 °C. Aliquots of 5 mL were withdrawn at intervals of 0.5, 1, 2, 4, 6, 12, and 24 h. The withdrawn samples were immediately replaced by equal volumes of the same medium. The drug content of the release samples was estimated spectrophotometrically at λ_max_ of 359 nm. The experiments were performed in triplicate.

### 2.9. Kinetic Evaluation of the Release Data

The data obtained from the in vitro release studies were analyzed using curve fitting to different kinetic models (zero order, first order, Higuchi diffusion model, and Korsmeyer–Peppas equation) [36]. The model that best described the data was selected based on the highest correlation coefficient (*R*^2^).

### 2.10. Ex Vivo Skin Permeation Study

Skin permeation studies of RT were carried out for the selected RT-NC hydrogel formulation (RT-NC2) and free RT hydrogel using the abdominal skin of a male mouse according to previously described procedures [37]. The study protocol was approved by The Research Ethics Committee, Faculty of Pharmacy, South Valley University, Egypt (approval number P.S.V.U 125/22). The animals were sacrificed, the dorsal hair was removed and the skin was cleaned three times with phosphate buffer pH 7.4. Fresh skin specimens were stretched over one end of the open-ended glass tubes with a total base surface area of 3.14 cm^2^ using an elastic rubber band. The tested gel formulations (1 g of free RT or RT-NC2 hydrogels equivalent to 5 mg of RT) were placed over the skin surface. The glass tubes were dipped in a glass beaker containing 100 mL of phosphate buffer (pH 6.5 with 0.25%, *v*/*v* ethanol). The beakers were shaken at 50 RPM and 37 ± 0.5 °C for 24 h in a thermostatic shaker water bath. At different time intervals (0.5, 1, 2, 4, 6, 12, and 24 h), samples of 5.0 mL were withdrawn, replaced with an equal volume of the fresh release medium, and analyzed spectrophotometrically at λ_max_ of 359 nm for RT content. The measurements were carried out in triplicate. The cumulative amount of drug permeated per unit surface area was plotted as a function of time. The slope of the linear regression line was taken as the steady state flux (*J_ss_*, µg·cm^−2^·h^−1^) [38]. The apparent permeability coefficient (*P*_app_, cm·h^−1^) was calculated using the following equation:

(4) $$P_{\mathrm{app}}=\frac{J_{ss}}{C_{0}}$$

where *C*_0_ is the initial concentration of RT (µg/mL) in the donor compartment.

### 2.11. In Vivo Anti-Inflammatory Paw Edema Studies

The acute anti-inflammatory activity for the selected hydrogel formulation was performed using a carrageenan-induced rat paw edema model [39]. The study protocol was approved by The Research Ethics Committee, Faculty of Pharmacy, South Valley University, Egypt (approval number P.S.V.U 125/22). The approximate weight of each rat was 200 g. The rats were randomly divided into four groups, each of four rats. Carrageenan (1%, *w*/*v*) in saline solution was injected subcutaneously into the left hind paw of the rats for the induction of edema. Group 1 received a placebo HPMC hydrogel and was used as an untreated control. Groups 2 and 3 received free RT and RT-NC2 hydrogels, respectively. Group 4 received a marketed diclofenac sodium gel 1% (Olfen^®^, Medical Union Pharmaceuticals, Cairo, Egypt) as a reference anti-inflammatory agent. The tested formulations were applied on the edematous paw 30 min post induction which was considered as the zero time of treatment. The growth in the paw thickness was determined using a vernier caliper. The measurements were performed in triplicate. The percent edema and percent edema inhibition were calculated using the following equations:

(5) $$\mathrm{Edema}\;\left(E,\;\%\right)=\frac{V_{t}-V_{0}}{V_{0}}\times 100\;$$

(6) $$\mathrm{Edema}\;\mathrm{inhibition}\;\left(\%\right)=\frac{E_{c}-E_{t}}{E_{c}}\times 100$$

where *V*_0_ and *V_t_* are the mean paw volume before and after carrageenan injection at time *t*, respectively. *E_c_* and *E_t_* are the edema percentages of control and treated groups at the same time interval, respectively.

### 2.12. Statistical Analyses

The experiments were run in triplicate and the results were represented as mean ± SD. GraphPad Prism software version 8.0.1 (GraphPad Software Inc., La Jolla, CA, USA) was used to statistically analyze the data. One-way analysis of variance analysis (ANOVA) with Tukey’s post-hoc test was used. A difference of *p* < 0.05 was predefined as statistically significant.

## 3. Results and Discussion

### 3.1. RT-NCs Preparation and Characterization

Despite the enormous advantages of nanocrystals including high drug loading and improved dissolution and saturation solubility, they suffer from poor physical stability that results from their small particle size and the associated increase in free energy leading to aggregation [40,41]. To enhance RT-NCs’ stability, various stabilizers were used in this study including nonionic surfactants such as Tween 80 and nonionic polymers such as Pluronic F127, HP-β-CD, and PEG 6000. They are believed to stabilize nanocrystals through adsorption on their surface forming protective layers against particle aggregation and crystal growth [41].

### 3.2. Particle Size, Polydispersity Index, and Zeta Potential Measurements

Table 2 shows the particle size, polydispersity index, zeta potential, and percent drug entrapment efficiency (%EE) for RT-NCs prepared using various stabilizers. The particle sizes ranged from 270.5 ± 16.7 to 505.8 ± 20.5 nm. The smallest size was detected for HP-β-CD RT-NCs while Tween 80 formed the largest particles with the differences being statistically significant at *p* < 0.05 except RT-NC1 versus RT-NC2. The particle size of nanocrystals is controlled by several factors, such as the method of preparation, eventual presence of stabilizers, and the type of stabilizer. The generation of nanocrystals is associated with an enormous increase in surface area due to the production of a large number of small particles and a vast decrease in particle size. This is associated with increasing the system Gibb’s free energy leading to thermodynamic instability [42]. These nanoparticles will eventually agglomerate in an attempt to minimize their total energy [43]. Stabilizers (e.g., surfactants and polymers) are thus required to minimize the system free energy and prevent agglomeration. A successful stabilizer should be able to control the particle growth during the production of uniform nanoparticles [42]. The larger size for Tween 80-stabilized nanocrystals might be related to their weak ability to sterically stabilize the nanoparticles, allowing them to grow in size during preparation. On the other hand, the smaller size detected for HP-β-CD-stabilized nanocrystals might be due to their ability to perfectly coat the newly formed nanoparticles which sterically stabilized them and prevented their aggregation and increase in size. These results are in agreement with previous work which showed a larger particle size of Tween 80-stabilized nanocrystals compared to those stabilized by HP-β-CD [41]. The PDI values were in the range of ~0.3 to 0.5, thus indicating the acceptable size distribution of the nanocrystals. A PDI value of 0.05 or smaller indicates a monodispersed population while heterogeneous nanoparticles have a PDI more than 0.7 [44].

The zeta potential was measured for all of the prepared rutin nanocrystal formulations due to its paramount importance for nanoparticle colloidal stability; it represents the electrostatic barrier that prevents nanoparticle aggregation and agglomeration [42,45]. Table 2 shows that the zeta potential of rutin nanocrystals ranged from −12.4 ± 1.0 to −28.8 ± 1.0 mV with the differences being statistically significant at *p* < 0.05 except RT-NC2 versus RT-NC3. RT-NCs had negative zeta potential values, probably due to the adsorption of water hydroxide ions at the nanocrystal surface [46,47]. It was previously shown that an absolute zeta potential value of around 30 mV is required for good colloidal stability [45]. However, this applies when the stabilization depends on pure electrostatic forces only without contributions from steric stabilization [42]. For instance, it was previously shown that nanosuspensions stabilized by non-ionic polymers and surfactants showed good colloidal stability while having zeta potential values much lower than the suggested value of 30 mV [28,41,48].

### 3.3. Percent Drug Entrapment Efficiency (%EE) Measurements

The percent drug entrapment efficiency (%EE) ranged from 65.7 ± 0.7% for RT-NCs prepared with Tween 80 to 75.5 ± 0.9% for those prepared with HP-β-CD. The differences were statistically significant at *p* < 0.05 except for those between RT-NC4 and either RT-NC1 or RT-NC3. The %EE was measured by an indirect method where the nanocrystals were separated by centrifugation and the drug content in the supernatant was measured. Thus, the highest %EE for HP-β-CD-stabilized nanocrystals is probably due to its ability to effectively coat and stabilize the nanoparticles which prevented their escape in the supernatant. On the other hand, the relatively lower %EE detected for Tween 80 (non-ionic surfactant) and Pluronic F127 (non-ionic polymer) might be due to their ability to partially solubilize the drug in water through micelle formation which might have facilitated its escape in the supernatant, thus decreasing the %EE [41,49].

In light of the above results RT-NCs with HP-β-CD as a stabilizer were selected for further studies since they showed the smallest particle size making them the most promising candidate to enhance rutin’s anti-inflammatory properties and penetration into the skin [18,25]. In addition, these RT-NCs also had the highest %EE of 75.5 ± 0.9%, thus limiting the needed excipients and maximizing the drug/excipient ratio. They also had the highest zeta potential of −28.8 ± 1.0 mV which suggests better colloidal stability compared with other RT-NC preparations.

### 3.4. Stability Studies

#### 3.4.1. Physical Stability of RT-NCs

The settlement volume ratio (*F*), the ratio between the volume or height of the nanocrystal suspension after and before sedimentation for a given period of time, is usually used as an indicator of nanocrystal suspension physical stability [31]. Table 3 shows that the *F* values were in the range of 0.15 to 0.95 with formulation RT-NC2 containing HP-β-CD showing the highest *F* values at all the studied time points. There was a general trend of a decrease in *F* values with time for all the studied preparations. At any time point, the *F* values followed this order: RT-NC2 (HP-β-CD) > RT-NC1 (Pluronic F127) > RT-NC3 (Tween 80) > RT-NC4 (PEG 6000). All the differences were statistically significant (*p* < 0.05). The lowest *F* values were detected for the nanocrystals with PEG 6000 as a stabilizer. Thus, a value of 0.21 ± 0.01 was measured for freshly prepared nanocrystals that gradually decreased to 0.15 ± 0.01 after three weeks indicating poor colloidal stability. In contrast, RT-NC2 containing HP-β-CD had the best colloidal stability as indicated by the highest *F* values among the tested preparations. A value of 0.95 ± 0.03 that was measured for freshly prepared samples decreased to 0.89 ± 0.05 after three weeks with no significant difference (*p* > 0.05). This high stability might be related to the relatively higher zeta potential of −28.8 ± 1.0 mV for HP-β-CD-stabilized nanocrystals in addition to their ability to sterically stabilize the nanocrystals. Similar high stability was previously observed for HP-β-CD-stabilized daidzein nanocrystals confirming its ability to efficiently coat the nanocrystals and prevent their agglomeration over time [41]. These results support the selection of formulation RT-NC2 for further studies.

#### 3.4.2. Storage and Photostability

To further characterize the stability of RT nanocrystals, the selected formulation (RT-NC2 stabilized by HP-β-CD) was stored at room temperature (25 °C) and refrigerated conditions (4 °C) for 60 days and their particle size, polydispersity index, and percent drug entrapment efficiency were determined. Table 4 shows that there was a gradual decrease in the percent drug entrapment efficiency with time. Thus, after 60 days of storage the %EE decreased from 75.53 ± 0.91 to 71.23 ± 1.07 and 70.00 ± 1.00 for the samples stored at 4 °C and 25 °C, respectively. The %EE after 60 days was significantly smaller than that of either zero time or 30 days of storage (*p* < 0.05). Furthermore, the storage temperature had no important influence on the %EE as evidenced by a non-significant difference between the samples stored at 4 °C and 25 °C. The small decrease in %EE over time might be attributed to the drug solubilization over time by HP-β-CD which converts the drug from a nanocrystal to a solubilized form and facilitates its escape to the surrounding bulk medium.

Regarding the particle size, there was a general size increase over time regardless of the storage temperature, albeit the increase at 25 °C was higher than at 4 °C. Thus, the size after 60 days of storage at 25 °C was significantly bigger compared to that of all other tested samples (*p* < 0.05). This indicates that storage in refrigerated conditions is advisable for these nanocrystals. A similar bigger particle size at a higher storage temperature was observed in other pieces of research and attributed to the increase in nanoparticle kinetic energy at higher temperatures leading to a higher probability of particle collisions and subsequently increasing the particle size [41,50,51]. Similarly, there was a general increase in the PDI values over time. However, the differences were not significant compared with the freshly prepared nanocrystals (*p* > 0.05).

**Figure 1:**
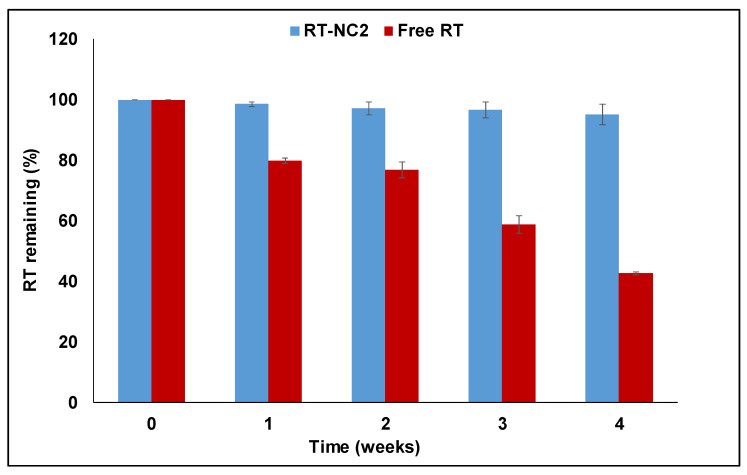
Percent remaining of RT as a function of exposure time to light for free RT and RT nanocrystals formulation RT-NC2.

Concerning RT photostability, previous studies have shown that RT is susceptible to photodegradation where exposure to UVB radiation for 120 min resulted in a decrease of 13.6% in RT content [52]. Our results show that light exposure caused progressive degradation of free RT (Figure 1). Thus, after 4 weeks of light exposure, only 42.7 ± 0.7% was remaining for free RT. In contrast, RT-NC2 had much better stability against light exposure. For instance, after the same time, the remaining RT for the nanocrystals was 95.1 ± 3.4%. This confirms that the nanocrystals stabilized by HP-β-CD had around 2.3-fold better RT photostability. This much better stability for the nanocrystals might be attributed to the protection effect offered by HP-β-CD where it covers the drug nanocrystals. These results are in agreement with previous reports showing better photostability of RT when formulated into nanoparticles [53].

### 3.5. FT-IR Spectroscopy Studies

**Figure 2:**
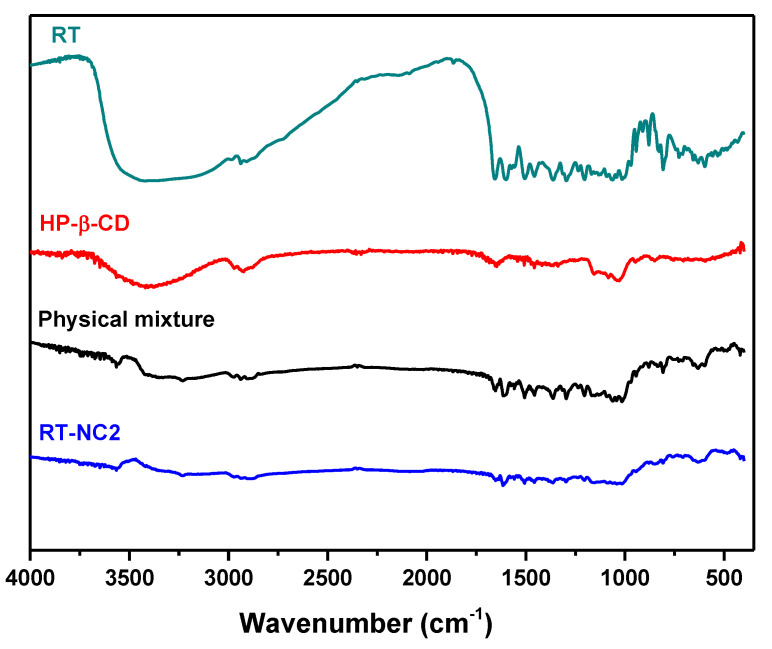
FT-IR spectra of rutin alone, HP-β-CD alone, their physical mixture (1:1, *w*/*w*), and HP-β-CD-stabilized nanocrystals (formulation RT-NC2).

The potential of chemical interactions between rutin and HP-β-CD in RT-NC2 was studied by recording the FT-IR spectra of rutin alone, HP-β-CD alone, their physical mixture (1:1, *w*/*w*), and the selected nanocrystal formulation (HP-β-CD-stabilized nanocrystals) and the results are shown in Figure 2. The spectrum of rutin alone shows a broad band centered at around 3430 cm^−1^ for OH bending, a sharp band at 1654 cm^−1^ due to C = O stretching, and a sharp band at 1594 cm^−1^ for C = C stretching of aromatic structures which is in agreement with published reports [54]. The spectrum of HP-β-CD alone shows a broad band centered at around 3400 cm^−1^ ascribed for vibration of free –OH groups and a band at 2927 cm^−1^ for vibration of bound -OH groups. The spectrum of the rutin/HP-β-CD physical mixture shows as sharp band at 1652 cm^−1^ ascribed to the stretching of rutin carbonyl groups while the stretching of rutin C = C of aromatic structures is slightly shifted to 1614 cm^−1^. These bands appeared at the same wavenumbers in the spectrum of RT-NC2 nanocrystals (1652 and 1614 cm^−1^, respectively) confirming the absence of chemical or physical interactions between rutin and HP-β-CD.

### 3.6. Saturation Solubility Measurements

**Figure 3:**
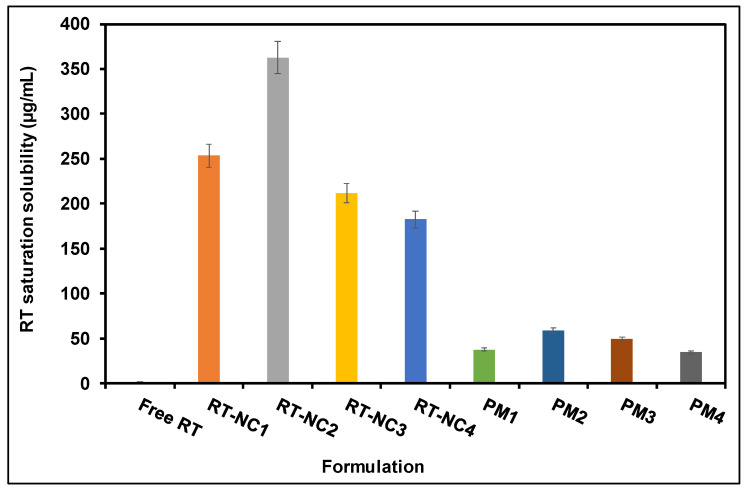
Saturation solubility of various RT-NCs in phosphate buffer pH 6.5 in comparison to free RT and various corresponding physical mixtures (PM). RT-NC1: rutin nanocrystals formulation 1, RT-NC2: rutin nanocrystals formulation 2, RT-NC3: rutin nanocrystals formulation 3, RT-NC4: rutin nanocrystals formulation 4.

RT is a hydrophobic compound with poor aqueous solubility which limits its bioavailability and clinical benefits [55,56]. The results obtained (Figure 3) show that RT solubility in phosphate buffer pH 6.5 was 1.8 ± 0.7 µg/mL. Rutin is a weak acid with *p*Ka in the range of 7.1 to 11.65 leading to a pH-dependent solubility profile [57]. Conversion of RT into NCs resulted in a significant increase in its aqueous solubility for all the tested stabilizers (Figure 3) (*p* < 0.05). For instance, NCs showed around a 102- to 202-fold increase in RT aqueous solubility that was dependent on the type of the stabilizer. The degree of solubility enhancement followed this descending order: HP-β-CD (RT-NC2) > Pluronic F127 (RT-NC1) > Tween 80 (RT-NC3) > PEG 6000 (RT-NC4). This might be related to the nanocrystal particle size where HP-β-CD-stabilized nanocrystals had the smallest particle size of 270.5 ± 16.7 nm among the tested stabilizers (Table 2). According to the Ostwald–Freundlich equation, the decrease in particle size results in increasing the particles’ surface area which in turn leads to increasing rutin’s aqueous solubility [58,59]. However, particle size is not the only factor influencing aqueous solubility. For example, Tween 80-stabilized nanocrystals had a bigger size than those stabilized by PEG 6000 but they had better solubility (Table 2). This is presumably attributed to the ability of Tween 80 to form micelles that encapsulate hydrophobic drugs such as rutin and increase their aqueous solubility [41,49].

The drug physical mixtures with the used stabilizers also achieved significantly higher drug aqueous solubility compared with the free drug hydrogel (*p* < 0.05) [60]. This might be attributed to the hydrophilicity of the used stabilizers which facilitates drug dissolution and solubility in water. In addition, the nanocrystals had significantly higher drug solubility compared with the corresponding physical mixture. This is probably due to the size reduction and increase in surface area achieved by the nanocrystals.

### 3.7. Drug Dissolution Studies

**Figure 4:**
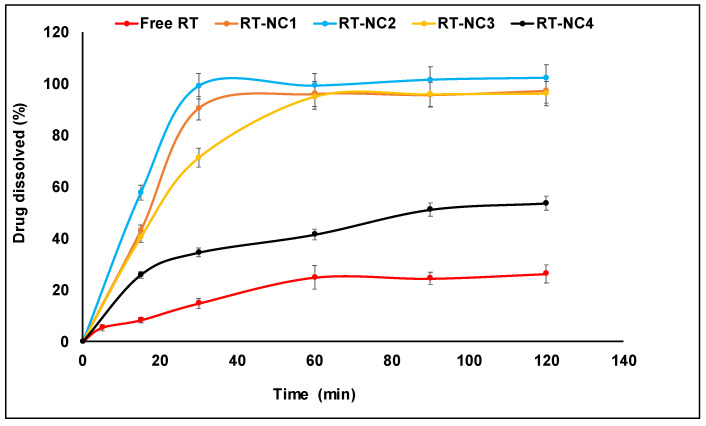
Dissolution profiles of various RT-NCs in phosphate buffer pH 6.5 at 37 °C in comparison to free RT.

Figure 4 shows the percent RT dissolved as a function of time for various nanocrystal formulations in comparison to the free drug. Free RT had the slowest dissolution rate among the tested preparations where around only 25% was dissolved after 120 min. RT is known as a hydrophobic compound with a slow dissolution rate which explains this slow dissolution [54]. Interestingly, the nanocrystal formulation RT-NC2 containing HP-β-CD as a stabilizer achieved 100% drug dissolution in 30 min compared with around only 15% for the free drug. Other nanocrystal formulations had significantly faster drug dissolution rates compared with the free drug (*p* < 0.05). However, except for RT-NC1, RT-NC2 had significantly faster drug dissolution compared with the other tested RT-NC formulations after 30 min (*p* < 0.05). After 30 min, the percent of drug dissolved followed this descending order: RT-NC2 > RT-NC1 > RT-NC3 > RT-NC4. Thus, they had 2.3-, 4.9-, 6-, and 6.7-fold higher dissolution rates compared with the free drug, respectively. This is the same order observed above for the saturation aqueous solubility and is probably attributed to the effect of particle size, surface area, and micelle formation on the drug dissolution rate. Previous studies have shown that the mechanism by which a given stabilizer enhanced the drug dissolution rate might have a more important influence compared with the particle size. Thus, etodolac nanocrystals’ dissolution rate was affected by the particle size, as well as the type of stabilizer [42]. The % etodolac dissolved for β-cyclodextrin-stabilized nanocrystals with a particle size of 866 nm was higher than that observed for Tween 80-stabilized nanocrystals with a particle size of 393 nm. This observation was attributed to the ability of β-cyclodextrin to form a water-soluble inclusion complex with etodolac which increased its dissolution rate [61]. The viscosity of the dissolution medium and its ability to influence the drug ionization status were also found to affect the drug dissolution rate [42].

### 3.8. SEM Observations

**Figure 5:**
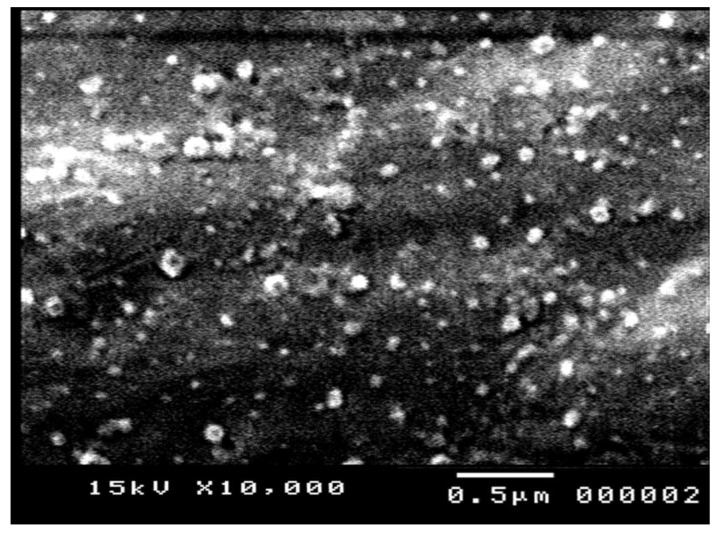
Scanning electron microscope photomicrograph of RT NCs prepared using HP-β-CD as a stabilizer (F2).

Figure 5 shows an SEM photomicrograph of RT-NCs prepared using HP-β-CD (RT-NC2). The nanocrystals appear as homogenously distributed spherical particles with distinctive boundaries and no aggregation. The size obtained from this measurement was 111.2 ± 29.5 nm. This size is smaller than that measured by DLS (270.5 ± 16.7 nm), probably due to the dry nature of the samples measured by SEM compared to the hydrated particles measured in DLS [62]. During sample measurement in SEM, the hydrated shell collapses during drying in the high-vacuum chamber of the SEM resulting in dried nanoparticles having a smaller particle size [63].

### 3.9. Characterization of Free RT and RT-NC2 Hydrogels

A hydrogel formulation was selected to facilitate RT-NC2 application on the skin since previous studies have shown that hydrogels were more efficacious than other vehicles in promoting drug–skin penetration from nanocrystal formulations [64,65]. Hydrogels have high water content, bioadhesive properties, and could serve as a depot system allowing for sustained drug delivery to the skin [66]. The properties of free RT and RT-NC2 hydrogels are shown in Table 5.

### 3.10. Drug Release Studies of Free RT and RT-NC2 Hydrogels

**Figure 6:**
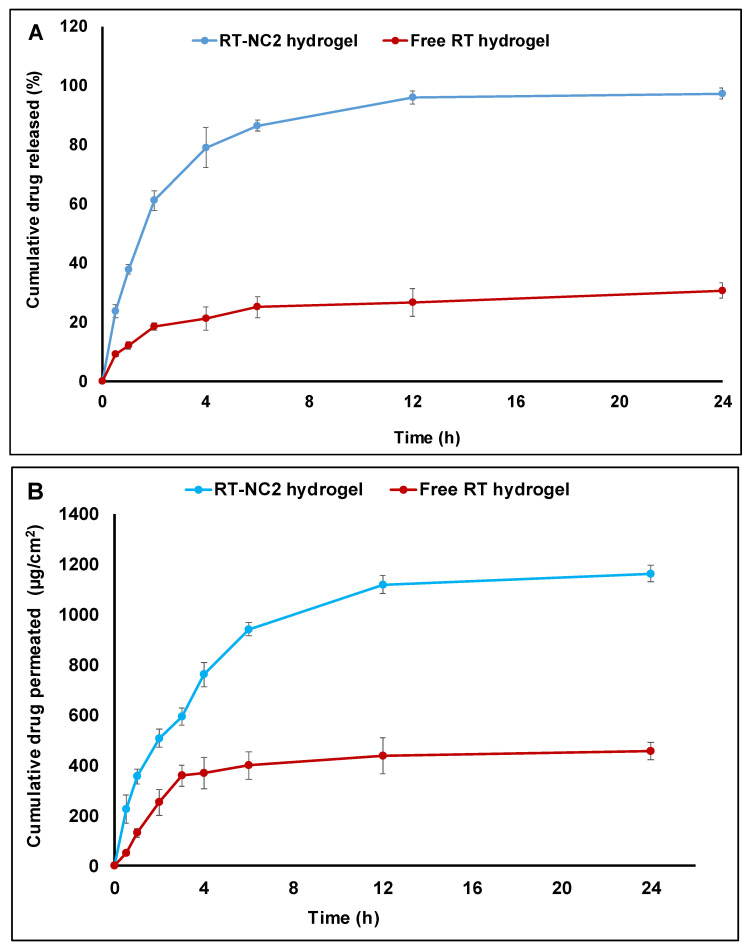
(**A**) Drug release profiles from RT-NC2 hydrogel in comparison to the free drug hydrogel in phosphate buffer pH 6.5 containing 0.25%, *v*/*v* ethanol at 37 °C. (**B**) Cumulative amount of RT permeated per unit surface area of mouse abdominal skin (µg/cm^2^) for free RT hydrogel and RT-NC2 hydrogel.

Figure 6A shows that drug release from the free drug hydrogel was slow whereby around only 30% of the drug was released after 24 h. This is presumably due to the hydrophobic nature of the drug which limits its dissolution rate and aqueous solubility. Release of a drug suspended in a hydrogel base is believed to include two steps: drug dissolution followed by diffusion of the solubilized drug through the hydrogel matrix. In contrast, a much faster drug release was observed for RT-NC2 hydrogel whereby almost complete drug release (~97%) was observed in 12 h. These results agree well with the enhanced dissolution rate and aqueous solubility described above for RT nanocrystals in comparison to the free drug. Similar behavior was also observed previously for nanocrystals suspended in a hydrogel base and was attributed to the small particle size of the nanocrystals leading to larger surface area and smaller diffusion distance and, hence, better drug dissolution and release [67]. The drug release medium was also reported to influence the drug release rate from nanocrystal formulations [42]. For instance, the pH of the release medium was found to affect the ionization status of ionic drugs leading to an important influence on their dissolution and release rate. Moreover, stabilizers that increase the release medium viscosity in the vicinity of a nanocrystal surface decreased the drug release rate from nanocrystal formulations [42].

**Figure 7:**
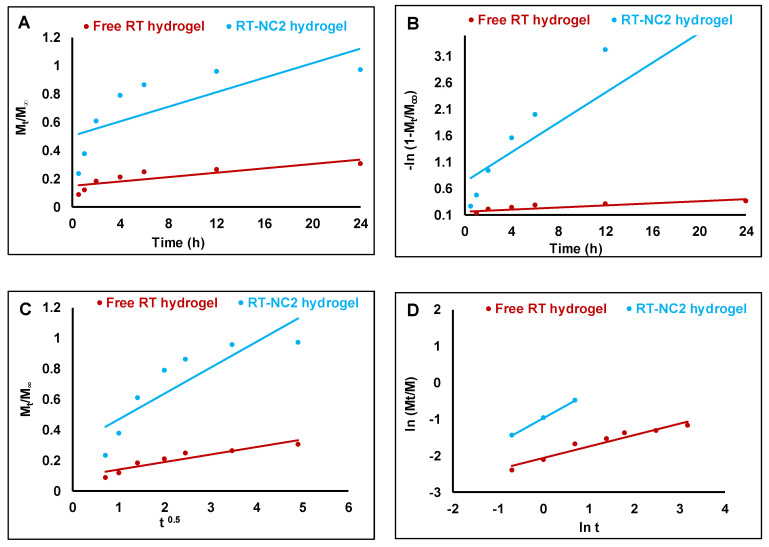
Plots of RT release data according to different kinetic models. (**A**) Zero order, (**B**) first order, (**C**) Higuchi diffusion model, (**D**) and Korsmeyer–Peppas equation.

The release data were analyzed using various mathematical models and the correlation coefficient (*R*^2^) was calculated to obtain insights into the drug release mechanism (Figure 7) [68]. The *R*^2^ values of free RT hydrogel were 0.74, 0.92, 0.87, and 0.97 for the zero order, first order, Higuchi, and Korsmeyer–Peppas models, respectively. In addition, the *R*^2^ values of RT-NC2 hydrogel were, respectively 0.84, 0.86, 0.93, and 0.99 for the zero order, first order, Higuchi, and Korsmeyer–Peppas models (Table 6). This indicates that the drug release from both preparations followed the Korsmeyer–Peppas model. The release exponent (n) which indicates the release mechanism was 0.316 and 0.688 for the free drug hydrogel and RT-NC2 hydrogel, respectively. This confirms that the release from free drug hydrogel was governed by Fickian diffusion (case I diffusional) while that from the RT-NC2 hydrogel was governed by anomalous (non-Fickian) transport [69].

### 3.11. Ex Vivo Skin Permeation Study

Figure 6B shows the cumulative amount of RT permeated through mouse abdominal skin for the selected RT-NC2 hydrogel in comparison to the free drug hydrogel. RT nanocrystal hydrogel had significantly higher drug skin permeation where the cumulative drug amounts permeated after 24 h were 456.7 ± 35.5 and 1163.9 ± 33.9 µg·cm^−2^ for the free drug hydrogel and nanocrystal hydrogel, respectively. This indicates that the nanocrystals achieved around a 2.5-fold enhancement in the amount of drug permeated through the skin. Furthermore, the flux (*Jss*) and apparent permeability coefficient (*P*_app_) of the nanocrystal hydrogel were similarly enhanced by around 2.8- and 3.2-fold in comparison to the free drug hydrogel (Table 7), respectively. Similar enhancement in drug skin permeability properties was previously observed in other studies and attributed to the small particle size, enhanced dissolution, and solubility of the nanosized drug particles in comparison to the coarse drug particles. In addition, the nanocrystals might have better adhesion to the skin due to their small particle size and increased contact area with the skin which creates a positive concentration gradient between the nanocrystals and skin and ultimately leads to enhanced drug permeability [65,70,71].

### 3.12. In Vivo Anti-Inflammatory Paw Edema Studies

**Figure 8:**
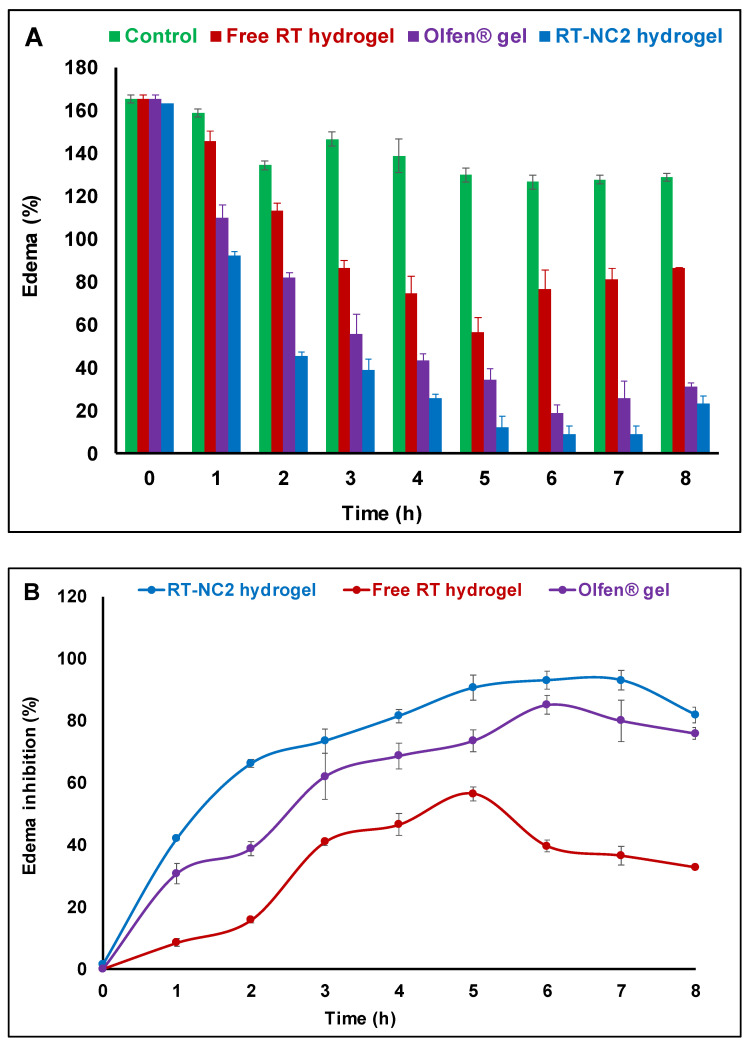
(**A**) Percent of paw edema as a function of time in rats after treatment with the selected rutin nanocrystal formulation (RT-NC2) hydrogel in comparison to rats treated with free RT hydrogel, Olfen^®^ gel, and untreated rats. (**B**) Percent of paw edema inhibition as a function of time in rats after treatment with the selected rutin nanocrystal formulation (RT-NC2) hydrogel in comparison to rats treated with free RT hydrogel and commercial Olfen^®^ gel.

The carrageenan-induced rat paw edema inflammatory model was used to assess the potential of HP-β-CD-stabilized RT-NCs hydrogel to enhance RT’s anti-inflammatory properties in comparison to untreated control, free RT hydrogel and diclofenac sodium commercial gel (Olfen^®^ gel) as a standard anti-inflammatory drug. The treatment was initiated 30 min post carrageenan injection and the percent edema was calculated (Figure 8A). The percent edema was highest at zero time for all of the tested preparations. Subsequently, there was a gradual decrease in the percent edema for all of the tested preparations. At any given time point, the percent edema followed this order: Control > free RT hydrogel > Olfen^®^ (diclofenac sodium) commercial gel > RT-NC2 hydrogel. All the differences were statistically significant (*p* < 0.05).

The percent edema inhibition was also calculated for the tested preparations and taken as a measure of their anti-inflammatory activity (Figure 8B). Both Olfen^®^ gel and RT-NC2 hydrogel achieved significantly higher percent edema inhibition compared with the free RT hydrogel at all the studied time points (*p* < 0.05). Peak edema inhibition was achieved at 7 h post administration for both Olfen^®^ gel and RT-NC2 hydrogel. At this time point, Olfen^®^ gel and RT-NC2 hydrogel had around 2.2- and 2.5-fold higher edema inhibition compared with free RT hydrogel, respectively. In addition, RT-NC2 hydrogel had significantly higher percent edema inhibition at all the studied time points except at 3 and 6 h compared with Olfen^®^ gel. The enhanced anti-inflammatory activity observed for RT-NC2 hydrogel could be explained on the basis of enhanced drug release and skin permeability (Figure 6) compared with the free drug hydrogel which might facilitate drug delivery to the inflammation site. Interestingly, the RT-NC2 hydrogel also had a better anti-inflammatory effect compared with the commercial diclofenac sodium gel (Olfen^®^ gel) which is presumably due to the nanometric particle size of the RT crystals which enhances drug dissolution and augments its penetration through deep skin layers and eventually results in a better anti-inflammatory effect. This finding is promising since a better anti-inflammatory effect is achieved by the RT-NC2 hydrogel than by the standard non-steroidal, anti-inflammatory drug diclofenac sodium without its notorious side effects which might increase patient compliance. In addition, site-specific drug delivery through topical application is expected to further improve drug safety and efficacy. Previous studies have shown that nanocrystal preparations were able to increase the anti-inflammatory properties of several other drugs [72,73,74].

## 4. Conclusions

Rutin nanocrystals were successfully prepared by the anti-solvent nanoprecipitation–ultrasonication method using various stabilizers such as non-ionic surfactants and non-ionic polymers. The type of stabilizer had a great influence on the nanocrystal properties. Thus, HP-β-CD gave the most favorable nanocrystal properties in terms of small particle size, high drug entrapment efficiency, high zeta potential, good colloidal stability, and the highest drug photostability. In addition, HP-β-CD-stabilized nanocrystals had around a 202- and 6.7-fold enhancement in drug aqueous saturation solubility and dissolution rate, respectively. HP-β-CD also affected the drug release rate and permeability through skin. Thus, HP-β-CD-stabilized rutin nanocrystals dispersed in HPMC hydrogel had around a 2.5-fold higher skin permeability than the free drug hydrogel. This better permeability resulted in an enhanced in vivo anti-inflammatory effect compared to the free drug hydrogel and commercial diclofenac sodium gel. Collectively, these results show the importance of the careful selection of nanocrystal stabilizers to optimize drugs’ physicochemical properties and maximize their in vivo efficacy.

## Figures and Tables

**Table 1:** Composition of RT-NCs prepared using different stabilizers at a weight ratio of 2:1, *w*/*w* (drug: stabilizer).

Ingredients	RT-NC1	RT-NC2	RT-NC3	RT-NC4
Rutin (mg)	30	30	30	30
Pluronic F-127 (mg)	60	-	-	-
HP-β-CD (mg)	-	60	-	-
Tween 80 (mg)	-	-	60	
PEG 6000 (mg)	-	-	-	60
PEG 200 (mL)	1	1	1	1
Ethanol (mL)	5	5	5	5
Water (mL)	30	30	30	30

**Table 2:** Particle size, polydispersity index, zeta potential, and percent drug encapsulation efficiency of various RT-NCs formulations.

Formulation	Stabilizer	Size (nm)	PDI	Zeta Potential (mV)	%EE
RT-NC1	Pluronic F127	289.0 ± 13.5	0.50 ± 0.03	−17.8 ± 0.5	68.4 ± 0.8
RT-NC2	HP-β-CD	270.5 ± 16.7	0.32 ± 0.02	−28.8 ± 1.0	75.5 ± 0.9
RT-NC3	Tween 80	505.8 ± 20.5	0.56 ± 0.07	−27.62 ± 1.1	65.7 ± 0.7
RT-NC4	PEG 6000	370.5 ± 17.9	0.51 ± 0.09	−12.4 ± 1.0	66.2 ± 0.8

All data are presented as mean ± SD.

**Table 3:** Settlement volume ratios for various RT nanocrystals after storage at room temperature for various time periods.

Time	Settlement Volume Ratio (*F*)			
	RT-NC1	RT-NC2	RT-NC3	RT-NC4
Freshly prepared	0.79 ± 0.02	0.95 ± 0.03	0.74 ± 0.03	0.21 ± 0.01
One week	0.77 ± 0.01	0.91 ± 0.01	0.72 ± 0.02	0.19 ± 0.02
Two weeks	0.75 ± 0.02	0.9 ± 0.03	0.69 ± 0.01	0.17 ± 0.01
Three weeks	0.72 ± 0.02	0.89 ± 0.05	0.68 ± 0.02	0.15 ± 0.01

**Table 4:** Effect of storage at room temperature (25 °C) and 4 °C on the percent drug entrapment efficiency (%EE), particle size (nm), and polydispersity index (PDI) of RT nanocrystals formulation RT-NC2.

Storage Temperature	Zero Time		30 Days		60 Days	
	4 °C	25 °C	4 °C	25 °C	4 °C	25 °C
%EE	75.5 ± 0.9	75.5 ± 0.9	73.6 ± 0.7	72.9 ± 1.2	71.2 ± 1.1	70.0 ± 1.0
Size (nm)	270.5 ± 16.7	270.5 ± 16.7	280.6 ± 5.4	275.6 ± 9.3	290.3 ± 7.8	320.4 ± 9.3
PDI	0.3 ± 0.02	0.3 ± 0.02	0.5 ± 0.02	0.4 ± 0.12	0.5 ± 0.13	0.4 ± 0.09

**Table 5:** Properties of free RT and RT-NC2 hydrogels.

Parameter	Drug Content	pH	Viscosity (cp)
Free RT hydrogel	95.92 ± 1.32%	6.8 ± 0.03	25,000 ± 45.1
RT-NC2 hydrogel	97.42 ± 1.1%	6.9 ± 0.01	45,263.33 ± 55.07

**Table 6:** Kinetic parameters of various models of RT release data from free RT and RT-NC2 hydrogels.

Kinetic Models	Zero Order		First Order		Higuchi Diffusion Model		Korsmeyer–Peppas		
	*k*_0_	*R* ^2^	*K*_1_	*R* ^2^	*K_H_*	*R* ^2^	*n*	*K_kp_*	*R* ^2^
Free RT hydrogel	0.777 ± 0.04	0.835 ± 0.06	0.010 ± 0.032	0.855 ± 0.07	4.918 ± 0.090	0.930 ± 0.012	0.316 ± 0.005	0.010 ± 0.0005	0.966 ± 0.002
RT-NC2 hydrogel	2.551 ± 0.125	0.741 ± 0.01	0.147 ± 0.028	0.915 ± 0.016	16.960 ± 0.786	0.867 ± 0.012	0.688 ± 0.063	0.106 ± 0.0004	0.999 ± 0.002

**Table 7:** Ex vivo permeation parameters of RT from free drug hydrogel and RT-NC2 hydrogel through mouse abdominal skin.

Parameter	*Q* ^a^	*Jss* ^b^	*P*_app_ ^c^ × 10^3^
Free RT hydrogel	456.7 ± 35.5	12.9 ± 1.2	2.3 ± 0.2
RT-NC2 hydrogel	1163.9 ± 33.9	36.5 ± 1.7	7.3 ± 0.3

^a^ Cumulative amount of RT permeated per unit area (µg·cm^−2^) after 24 h. ^b^ Flux (permeation rate constant) at steady state (µg·cm^−2^·h^−1^), obtained from the slope of the regression line after plotting the cumulative amount of RT permeated per unit area vs. time. ^c^ Apparent permeability coefficient (cm·s^−1^) calculated from Equation (4).

## Data Availability

Not applicable.

## Abbreviations

Hydroxypropyl beta-cyclodextrin (HP-β-CD), liquid crystalline nanoparticles (LCNs), nanocrystals (NCs), rutin (RT), hydroxypropyl methyl cellulose (HPMC), rutin nanocrystals (RT-NCs), polydispersity index (PDI), percent drug entrapment efficiency (%EE), settlement volume ratio (*F*), Fourier transform infrared spectroscopy (FT-IR), scanning electron microscopy (SEM), one-way analysis of variance (ANOVA), physical mixtures (PM), correlation coefficient (*R*^2^), release exponent (n), flux (*Jss*), and apparent permeability coefficient (*P*_app_).
